# Effects of microplastics, pesticides and nano-materials on fish health, oxidative stress and antioxidant defense mechanism

**DOI:** 10.3389/fphys.2023.1217666

**Published:** 2023-06-26

**Authors:** Udayadharshini Subaramaniyam, Rethi Saliya Allimuthu, Shanu Vappu, Divya Ramalingam, Ranjini Balan, Biswaranjan Paital, Niranjan Panda, Prasana Kumar Rath, Nirmaladevi Ramalingam, Dipak Kumar Sahoo

**Affiliations:** ^1^ Department of Biochemistry, Biotechnology and Bioinformatics, Avinashilingam Institute for Home Science and Higher Education for Women, Coimbatore, India; ^2^ Redox Regulation Laboratory, Department of Zoology, College of Basic Science and Humanities, Odisha University of Agriculture and Technology, Bhubaneswar, India; ^3^ Department of Animal Nutrition, College of Veterinary Science and Animal Husbandry, Odisha University of Agriculture and Technology, Bhubaneswar, India; ^4^ Department of Veterinary Pathology, College of Veterinary Science and Animal Husbandry, Odisha University of Agriculture and Technology, Bhubaneswar, India; ^5^ Department of Veterinary Clinical Sciences, College of Veterinary Medicine, Iowa State University, Ames, IA, United States

**Keywords:** microplastics, fish oxidative stress, nano particles, nano-therapeutics, pesticides, signaling pathways, water contamination

## Abstract

Microplastics and pesticides are emerging contaminants in the marine biota, which cause many harmful effects on aquatic organisms, especially on fish. Fish is a staple and affordable food source, rich in animal protein, along with various vitamins, essential amino acids, and minerals. Exposure of fish to microplastics, pesticides, and various nanoparticles generates ROS and induces oxidative stress, inflammation, immunotoxicity, genotoxicity, and DNA damage and alters gut microbiota, thus reducing the growth and quality of fish. Changes in fish behavioral patterns, swimming, and feeding habits were also observed under exposures to the above contaminants. These contaminants also affect the Nrf-2, JNK, ERK, NF-κB, and MAPK signaling pathways. And Nrf2-KEAP1 signalling modulates redox status marinating enzymes in fish. Effects of pesticides, microplastics, and nanoparticles found to modulate many antioxidant enzymes, including superoxide dismutase, catalase, and glutathione system. So, to protect fish health from stress, the contribution of nano-technology or nano-formulations was researched. A decrease in fish nutritional quality and population significantly impacts on the human diet, influencing traditions and economics worldwide. On the other hand, traces of microplastics and pesticides in the habitat water can enter humans by consuming contaminated fish which may result in serious health hazards. This review summarizes the oxidative stress caused due to microplastics, pesticides and nano-particle contamination or exposure in fish habitat water and their impact on human health. As a rescue mechanism, the use of nano-technology in the management of fish health and disease was discussed.

## 1 Introduction

Fish account for more than 40% of all vertebrates, which varies in size, shape, habitat, and biology. Fish is a staple and affordable food source, rich in high-quality animal protein (Maulu et al., 2021a). Most people prefer fish to red meat and poultry because of its tenderness and digestibility. Fish is the source of eight essential amino acids, vitamins A, D, B1, B2, and B3, and fresh fish contains a trace amount of vitamin C. They are an excellent source of calcium and fluorine, essential for bone and teeth development in children (Paital, 2018a). Other highly bioavailable minerals in fish are phosphorus, magnesium, iodine, iron, zinc, and selenium (Thilsted et al., 2016; Maulu et al., 2021a; Maulu et al., 2021b; Ahmed et al., 2022). The foods contain a significant amount of omega-3 fatty acids, such as EPA (eicosapentaenoic acid) and DHA (docosahexaenoic acid). About 140 gm of fish can fulfill the requirement of daily protein (50%–60%) consumption in an adult. All these nutrients keep a person healthier and free from cardiovascular, neurodegenerative, and low blood pressure (Balami et al., 2019; Kwasek et al., 2020). Significantly EPA and DHA help improve children’s cognitive development during pregnancy and prevent preterm birth.

People’s interest in fish production and consumption has increased recently because of their nutritional benefits (FAO, 2016; FAO, 2020). According to FAO, 2020, approximately 179 million tons of fish were produced globally in 2018. Of this, humans consumed around 156 million tons, accounting for 87% of the total fish production. The remaining 12% accounts for non-food products such as fishmeal and oil production. A survey by Ngasotter et al. (2020) indicates that India is ranked third in overall fish production in the world. According to Hodar et al. (2020), the overall fish production in India is roughly 12.60 million metric tonnes, with approximately 65% of that coming from inland sources. Additionally, about 50% of the total production is attributed to cultured fish. The growth and health of fish depend on the water quality in which they are cultured. Fish growth and production can be affected by various physical (temperature and solid concentrations), chemical, or biochemical (hardness, pH, and alkalinity) changes (Paital and Chainy, 2016; Paital and Rivera-Ingraham, 2016). Besides the above factors, some infectious diseases and pollutants, such as pesticides, microplastics, and even nanoparticles, also affect fish growth, production, reproduction and disease susceptibility or resistance (Paital, 2018c; Wu et al., 2020).

Recent studies estimate that about 5–13 million tons of plastic enter the oceans annually (Burgos-Aceves et al., 2022; Narra et al., 2022). Because of their small size, aquatic organisms like bivalves, fish, zooplankton, shrimp, and whales ingest microplastics (Guzzetti et al., 2018; Harmon, 2018; Rezania et al., 2018; Alimba and Faggio, 2019; Prokić et al., 2019; Strungaru et al., 2019). Microplastics are found as sediments or fragments of various polymers in the aquatic environment. Microplastics have been traced to 728 fish species worldwide (Hossain and Olden, 2022). After ingestion, microplastics accumulate in the gastrointestinal tract (GI) and block digestive system of fish, including the stomach and intestine, which reduces their feeding ability (Wright and Kelly, 2017). Microplastics adhere to fish skin, translocate to tissues like gills, muscles, and liver, and enter the circulatory or lymphatic system, causing nutritional and growth disorders (Lusher et al., 2017). Microplastic exposure induces fish behavioral changes, including altered feeding, swimming, weakened predatory performance, foraging, and ventilation (Liang et al., 2023). Microplastic ingestion affects immunity, growth, reproduction, survival, metabolism, and other toxicity responses (e.g., oxidative stress) in fish. Additionally, microplastics can cause organ damage, inflammatory responses, and apoptosis. Microplastics are highly concentrated in the digestive tracts of small fish and bivalves. On consumption of those fish, microplastics enter the human diet (Smith et al., 2018).

Another major contaminant in the aquatic ecosystem is pesticides. The freshwater system can be contaminated with pesticides via various means, such as wastewater discharge, spray drifts, agricultural runoff, rural spillovers, and leaching. Due to the bioaccumulation of toxic substances in various tissues and organs of fish, the concentration of pesticides in marine organisms is several times higher than in the ecosystem (Kaur and Jindal, 2017). Aquatic ecosystems have been negatively affected by the heightened levels of pesticide residues found in water and sediments, resulting in significant biodiversity loss. Pesticides can directly affect fish. Small fishes are affected more than larger ones. Pesticides also have indirect toxic effects on the life of aquatic organisms and food sources such as plankton and algae, and that leads to worsening the purity of aquatic organisms (Hassaan and El Nemr, 2020). Initially, pesticides enter through fish’s skin, gills, and digestive systems, pass many biological membranes, and accumulate in tissues. Some pesticides are metabolized in the body and get eliminated with the help of body fluids such as urine. Pesticide accumulation causes neurophysiological and hematological damage, endocrine disruption, DNA damage, apoptosis, and lipid peroxidation, leading to oxidative stress. Pesticide exposure also affects growth, reproduction (Sánchez et al., 2021), behavior (Sharma et al., 2019), and swimming performance (Yang et al., 2020; Atamanalp et al., 2021) in fishes.

**FIGURE 1:**
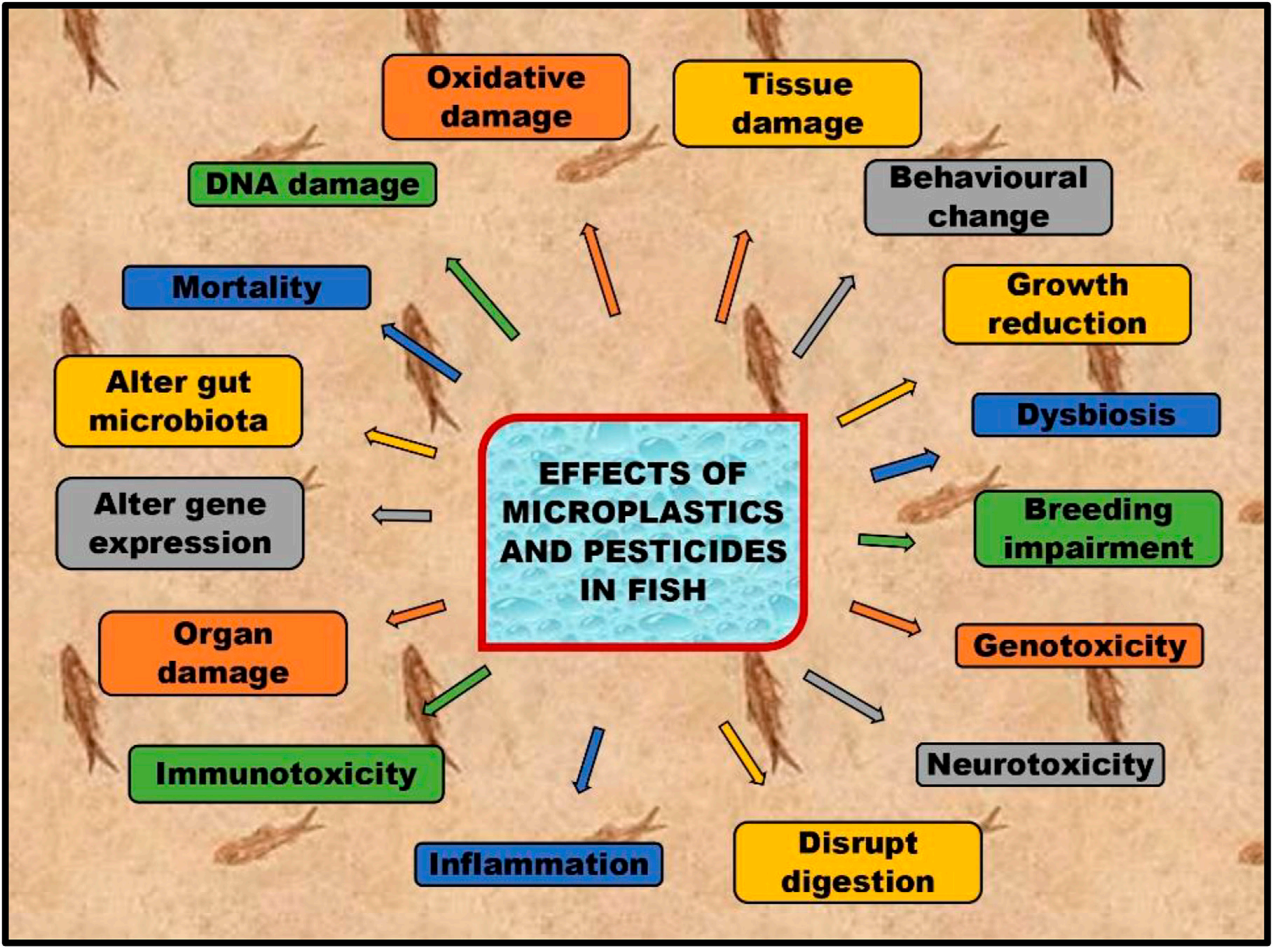
Effects of microplastics and pesticides on fish.

Microplastics and pesticides affect human health due to their accumulation in seafood (Nicole, 2021; Burgos-Aceves et al., 2022. Aquatic organisms ingest and retain microplastics and pesticides in some organs, causing oxidative stress, thus reducing the growth and quality of sea animals (Burgos Aceves et al., 2018a; Burgos Aceves et al., 2018b; Ngasotter et al., 2020; Pagano et al., 2020; Stara et al., 2020). Oxidative stress is a phenomenon that occurs when the body’s reactive oxygen species (ROS) and antioxidants are not balanced between each other (Bhatti et al., 2018). ROS are molecules produced naturally or induced in the body during metabolic processes, but their levels can increase due to external factors such as exposure to pollution or toxins. When ROS levels are high, they can cause damage to cells and tissues, leading to inflammation and other adverse health effects. A decrease in fish nutritional quality and population has a significant impact not only on the human diet but also influence traditions and economics worldwide (Liang et al., 2023). This review focuses on oxidative stress caused due to microplastics and pesticide contamination in fish and their impacts on human health (Figure 1).

It can be summarized that the stress induced by various environmental contaminants, including microplastics and pesticides, clearly induce various stresses out of which the induction of oxidative stress is emphatically studied (Cole et al., 2011; Bhatti et al., 2018; Ngasotter et al., 2020; Cole et al., 2021; Liang et al., 2023). Various nano-particles, although found to generate ROS in fish (Paital et al., 2019), nano-technology is also used to be helpful for the health management in fish. Some of the nano-particles also seem useful in managing fish diseases (Sahoo and Chatli, 2015; Stara et al., 2019; Yin et al., 2019; Liu et al., 2020; Kim et al., 2021; Pei et al., 2022; Xiao et al., 2023). Therefore, this review focused on how pesticides and microplastics induce the accumulation of ROS and oxidative and other stress in fish and their health management by use of nano-technology.

## 2 Oxidative stress and antioxidant defense mechanism

About 5% of oxygen consumed by the animals is eventually converted into ROS under normal conditions. The conversion of the respired oxygen to ROS follows a specific pathway that involves both electron transport chain (ETC) and oxidative phosphorylation. During the transport of electrons via ETC, some (about 5%) of them are leaked to the inter-membrane space of mitochondria at the complex I and III enzymes. The leaked electrons reduce oxygen incompletely to produce active oxygen intermediates (Sies, 2015). Superoxide anion is the first product of the one-electron reduction of oxygen and, consequently, the second, third^,^ and fourth electron reduction products of oxygen as hydroxyl radical, hydrogen peroxide (H_2_O_2_) and water. The production of the above ROS occurs when oxygen undergoes either the first, second^,^ or third reduction by the electrons in that get leaked from ETC. These ROS can cause non-specific oxidation of various biomolecules, including lipids, proteins, and nucleic acids, leading to the formation of lipid peroxides, protein carbonyls, and nucleic acid adducts, respectively. This phenomenon has been extensively studied in aquatic organisms, including fish (Bal et al., 2022b). Out of all the produced ROS, H_2_O_2_ is required in minimal amounts for various signal transduction processes, while when its level increases, alone or in combination with the other ROS, it/they damages the biomolecules. Under normal physiological conditions, the generated ROS is neutralized by the antioxidant defense molecules, but under any physiological disturbance state, when more damage is incurred by ROS, a condition arrives called oxidative stress. Since oxidative stress is the outcome of the damage to major macromolecules, it leads to additional trouble for animals, including increased disease susceptibility, compromised immunity, lower rate of survivability, faster aging, etc. This mechanism is studied in many animal models, including fishes (Bal and Paital, 2023). As stated earlier, the defense mechanism, which includes enzymatic and non-enzymatic antioxidant molecules, can protect the cells of animals from oxidative stress.

### 2.1 Enzymatic

**FIGURE 2:**
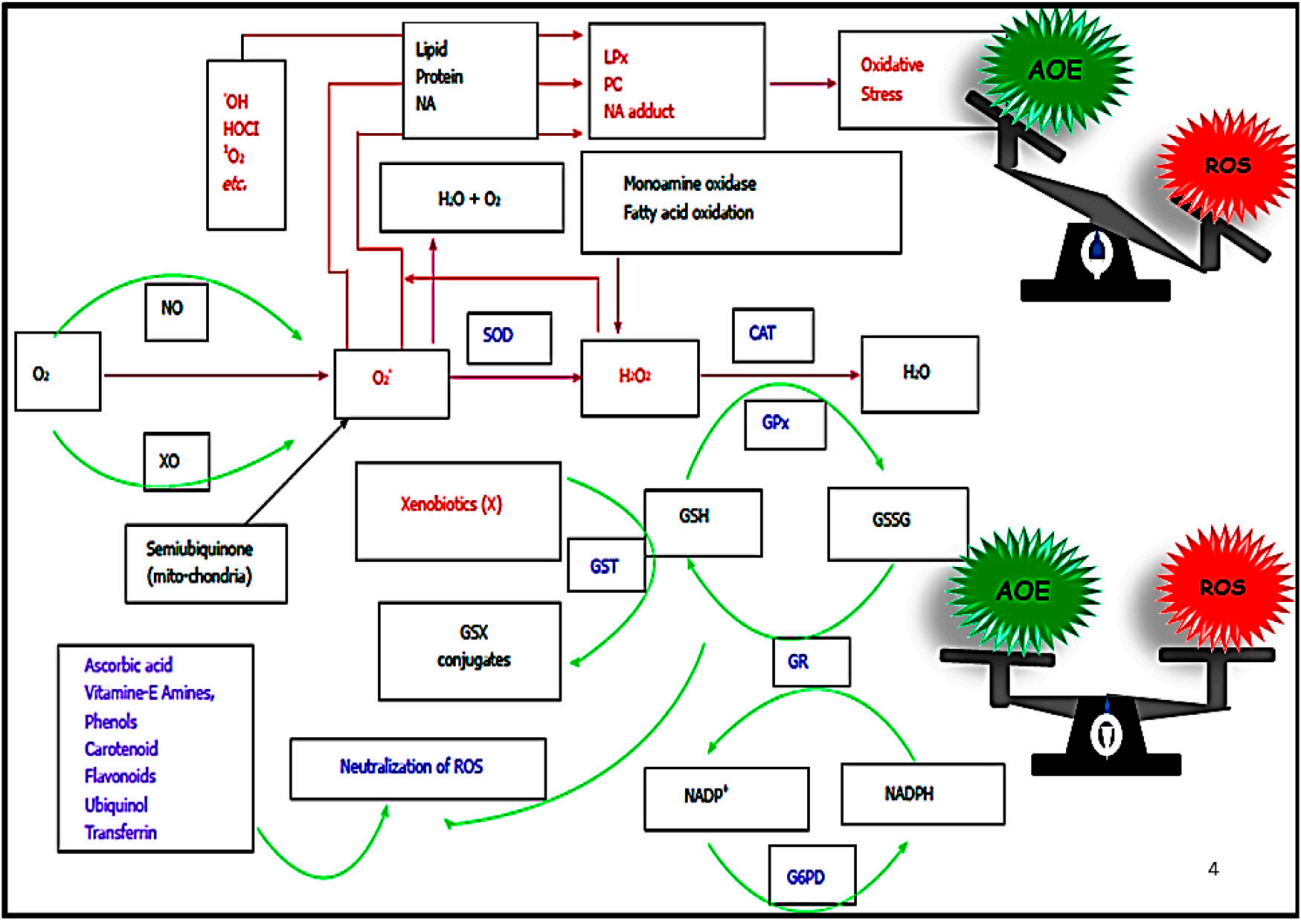
Oxygen acts as the precursor for the generation of ROS. Superoxide radical (O_2_˙^─^) is produced with the first electron reduction of oxygen. The produced O_2_˙^─^ is converted into H_2_O_2_ by the enzyme superoxide dismutase (SOD), H_2_O_2_ or hydroperoxides are neutralized by the enzyme catalase (CAT) or glutathione peroxidase (GPx), respectively. GPx uses one molecule of the reduced glutathione (GSH) by the process, and the produced oxidised glutathione (GSSH) gets back to GSH by the enzyme glutathione reductase (GR). GR uses one molecule of NADPH in the process and NADP^+^ produced by the process is reduced back to NADPH by the enzyme Glucose-6-phosphate dehydrogenase (G6PD). The enzyme glutathione-S-transferase (GST) also neutralizes xenobiotics with the help of GSH. Small antioxidants such as ascorbic acid, vitamins A, E, etc., can directly neutralize ROS non-specifically (modified after Paital, 2018b).

The enzymatic defense constitutes an array of enzymes that neutralize various specific ROS functions (Sies, 2015). Superoxide dismutase (SOD), the first line of enzymatic defense, dismutates superoxide radicals to H_2_O_2_ and H_2_O. The produced H_2_O_2_ is subsequently neutralized by another enzyme called catalase (CAT). The organic hydroperoxides are outraised by an enzyme called glutathione peroxidase (GPx). Water molecule is released in the process. For the action of GPx, a reduced glutathione (GSH) is consumed, which in turn is converted into oxidized glutathione (GSSG). The GSSG is then returned to its reduced form (GSH) by the enzyme glutathione reductase (GR). Glutaredoxin system also uses the reduction state of the glutathione to preserve and control the cellular redox cellular homeostasis and redox-dependent signaling pathways (Figure 2). The Keap1-Nrf2-ARE ((Kelch-like ECH-Associating protein 1) nuclear factor erythroid 2 related factor 2-antioxidant response element) regulates the signaling pathways for the transcription of the antioxidant enzymes (Hybertson et al., 2011; Mishra et al., 2019; Huang et al., 2021; Li et al., 2021; Paital et al., 2022).

### 2.2 Non-enzymatic

Unlike the enzymatic redox regulatory/antioxidant molecules, several non-enzymatic antioxidant molecules can directly and non-specifically neutralize the chemical nature of various ROS. These antioxidants are usually dietary in source in higher animals, whereas in lower animals and in some of the fishes, small antioxidants such as ascorbic acid are synthesized (Jones, 2008). The capacity for synthesis of ascorbic acid is lost in evolution in higher animals because of the absence of the enzyme L-Gulonolactone oxidase or L-Gulonolactone synthase. Besides ascorbic acid (vitamin C), other small non-enzyme antioxidants are GSH, vitamin A (retinol, β-carotene), vitamin E (tocopherol), polyphenolic compounds, etc. It is to be noted that all the above antioxidants work alone or in cascade to protect the cell from the toxic action of ROS, failing to which the cell experience oxidative stress, which is evident in animals, including fishes (Bal et al., 2022a; Bal et al., 2022b). Any external or internal stress including exposure to pollutants, toxicants, chemicals, microplastics, food availability, etc also generate more ROS to develop oxidative stress in fishes (Thakur et al., 2022). It is to be noted that higher levels of oxidative stress can hamper growth, production, reproduction, etc in animals, including fish. Therefore, the above factors can be considered to monitor the health status of (aquatic) animals in general and fish in particular under any stress, including microplastics, pesticides, and toxic nano-particles (Panda et al., 2022a; Panda et al., 2022b; Pati et al., 2022a; Pati et al., 2022b; Panda et al., 2023).

Oxygen acts as the precursor for the generation of ROS. Superoxide radical (O_2_˙^─^) is produced with the first electron reduction of oxygen. The produced O_2_˙^─^ is converted into H_2_O_2_ by the enzyme superoxide dismutase (SOD), H_2_O_2 or_ hydroperoxides are neutralized by the enzyme catalase (CAT) or glutathione peroxidase (GPx), respectively. GPx uses one molecule of the reduced glutathione (GSH) by the process, and the produced oxidised glutathione (GSSH) gets back to GSH by the enzyme glutathione reductase (GR). GR uses one molecule of NADPH in the process and NADP + produced by the process is reduced back to NADPH by the enzyme Glucose-6-phosphate dehydrogenase (G6PD). The enzyme glutathione-S-transferase (GST) also neutralizes xenobiotics with the help of GSH. Small antioxidants such as ascorbic acid, vitamins A, E, etc., can directly neutralize ROS non-specifically (modified after Paital, 2018b).

## 3 Microplastics induced oxidative stress in fishes

Microplastics are tiny plastic particles that are less than 5 mm in size. Detecting microplastics in micro and macro ecosystems is a growing environmental concern due to their persistence, ability to accumulate in ecosystems, and potential harm to aquatic and human health (Rochman et al., 2019). Based on their size, primary and secondary microplastics are of their two main categories. Primary microplastics are manufactured as tiny beads or fibers for various products. Secondary plastics enter the ocean via littering, improper disposal of plastic waste, wear and tear of plastic products, discharge of wastewater from households, industries, wastewater treatment plants, and accidental spills from plastic pellets during transportation and handling in industries (Li et al., 2020; Bakir et al., 2022). Microplastics enter aquatic environments and cause various damages to many marine organisms (Cai et al., 2021).

In fish, oxidative stress can be caused by various factors, such as exposure to pollutants, pathogens, and environmental stressors (Zhan et al., 2020). Excessive ROS production or impaired antioxidant defenses can cause oxidative stress, linked to various health effects in fish, including tissue damage, organ dysfunction, and impaired immune function (Padmini and Udayakumar, 2021). The exact mechanism by which microplastics cause oxidative stress may include the type, size, and shape of the plastic and the fish’s species and life stage having different levels of antioxidant levels (Figure 2). Liu et al. (2019); Liu et al. (2019b) found that microplastics act as carriers for bacterial pathogens and induce oxidative stress in common carp by surplus ROS generation with a decrease in antioxidant enzyme activity level; Yu et al. (2020); Yu et al. (2020b) found that nylon microplastic exposure causes the gills of crucian carp to experience oxidative stress by disrupting mitochondrial function and increasing the production of ROS. Similarly; Chen et al. (2021) found that polyethylene microplastics caused oxidative stress in the liver of tilapia by damaging cell membranes and increasing lipid peroxidation. Microplastic contamination in three economically valuable fishes, namely sea dace (Dicentrachus labrax), Atlantic horse mackerel (Trachurus trachurus), and Atlantic chub mackerel (Scomber colias), was studied by Barboza et al. (2020). The research found that microplastics were present in the dorsal muscle, gastrointestinal tract, and gills of 49% of the analyzed fish. Microplastic-contaminated fish had increased lipid peroxidation (LPO) levels in the brain, dorsal muscle, and gills and higher brain AChE activity than control fish groups. It was observed that 32% of the fish had microplastics in the dorsal muscle. The study revealed that an average human consumption of these three fish might intake 842 microplastic items yearly. Fang et al. (2021) reported that exposure to acrylic microplastics caused oxidative damage in the liver of crucian carp. The researchers found that ROS and LPO levels were increased while levels of antioxidant enzymes (CAT and SOD) were decreased. Li et al. (2020) revealed that exposure to polypropylene microplastics increased oxidative stress in the grass carp intestines by inducing inflammation and immune activation.

Microplastics can harm fish’s health, including gut, liver, and kidney damage. Studies show that when fish ingest microplastics, the particles can deposit in their organs and tissues, causing various adverse health effects (Dawson et al., 2018). Zhang et al. (2021) investigated the impact of polycarbonate microplastics on the liver and gut of grass carp. The researchers found that ingestion of microplastics resulted in significant damage to the liver, including increased lipid accumulation, fibrosis, inflammation, structural damage, and apoptosis. Zhang et al. (2020) found that polystyrene microplastic exposure caused liver and kidney damage in tilapia. The researchers observed changes in the morphology and function of these organs, as well as increased oxidative stress and inflammation. Furthermore, Wang et al. (2019) studied the exposure of polyurethane microplastics in zebrafish. They found that microplastics altered the gut microbiota composition, leading to oxidative stress and inflammation in the fish.

**FIGURE 3:**
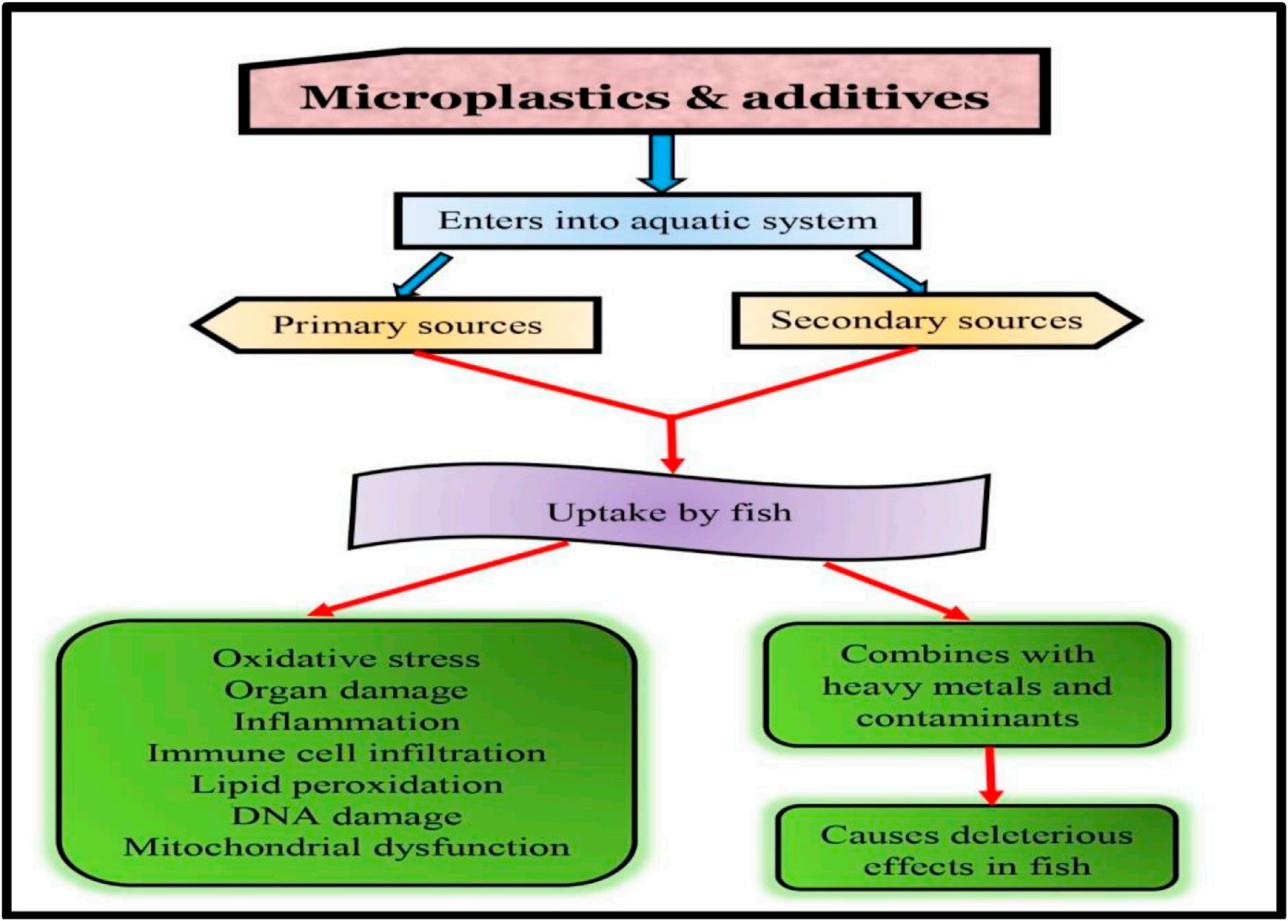
Microplastics in fish health.

In addition to causing organ damage and oxidative stress, microplastic ingestion can lead to changes behavioral patterns in fish. Lönnstedt and Eklöv. (2016) found that exposure to polyethylene terephthalate microplastics altered juvenile perch behavior. The researchers observed that the fish exposed to microplastics were more likely to exhibit risky behavior, such as swimming in open water, than the control fish. In addition to feeding and swimming behavior, microplastics can also affect the activity patterns of fish. Wright et al. (2020) found that exposure to polyvinyl chloride microplastics led to reduced swimming activity in brown trout and changes in their circadian rhythms. These behavioral changes can significantly affect fish’s survival and reproductive success and their role in the ecosystem. For example, reduced feeding rates and activity levels can lead to decreased growth and reproduction, while altered behavior can affect predator-prey interactions and community dynamics. Microplastic exposure can also impart a negative impact on reproductive success and survival rates in fish. Various research have revealed that microplastic ingestion can interfere with the reproductive systems of fish, leading to reduced fertility and hatching success and increased mortality rates (Figure 3).

### 3.1 Plastic additives in fish health

Plastic additives are tiny plastic particles or stabilizers added to various consumer products, such as personal care products, cleaning agents, and cosmetics, to provide specific properties or functions (Koelmans et al., 2019; Chokwe and Okonkwo, 2019). These additives can be composed of various plastics, such as polyethylene, polypropylene, and polystyrene, and can take different forms, including microbeads, microfibers, and microgranules (Ramachandraiah et al., 2022). Plastic additives can enter the environment through various pathways, including wastewater discharge, stormwater runoff, and accidental spills (Yusuf et al., 2022). Marine animals can also ingest plastic additives and accumulate in the food chain, a hazard to human health (Rochman et al., 2019). People who regularly eat seafood may ingest up to 11,000 microplastic particles annually (Cox et al., 2019). Plastic additives can cause oxidative stress in fish through a variety of mechanisms. One such mechanism is the release of chemicals from the microplastics that can disrupt the balance between levels of ROS and antioxidants in the fish (Chae and An, 2020). Another mechanism is the physical damage to fish tissues, leading to inflammation and increase in ROS production (Su et al., 2020).

Plastic additives ingestion can cause damage to fish organs, including the liver, kidney, and gut. Plastic exposure causes stress on the liver cells of zebrafish embryos and crucian carp (Li et al., 2018; Yang et al., 2020). Additionally, plastics have been exposed to accumulate in the gut of fish, which can lead to physical damage and inflammation (Brennecke et al., 2016). Fish that have ingested plastic additives can also exhibit behavioral changes. Chen et al. (2020) and Qiang and Cheng, 2019 found that plastic additives resulted in alterations in the swimming behavior of zebrafish and juvenile yellow perch feeding behavior, respectively. These behavioral changes can negatively impact fish survival and reproduction, as they change the behavior of fish for reproduction (mating), to avoid predators, and to find food. Plastic additives affect the immune system of fish by overexpressing the immune-related genes in zebrafish and Japanese medaka (Huang et al., 2021a; Liu et al., 2022).

According to Adeleye et al. (2014), plastic additives may contain additional water pollutants that can be additive factors to cause fish oxidative stress. Studies have indicated that microplastic additives have the ability to induce oxidative stress in fish, as demonstrated by several research studies. For instance, Boháčková et al., 2023 observed that rainbow trout exposed to plastic additives experienced a noteworthy increase in oxidative stress biomarkers. Another study by Wang et al., 2019 found that exposure to plastics led to oxidative damage in the liver tissues of zebrafish. Plasticizers are plastic additives that can be liberated into the environment under these circumstances; Iheanacho et al. (2020) exposed *Clarias gariepinus* to plastic additives (melamine formaldehyde, polyvinyl chloride, melamine) and observed the generated neurotoxicity and oxidative stress in the exposed fish as compared to the control groups. In addition, the fish experienced liver damage, as indicated by increased levels of AST and ALT and hypoproteinemia. Dietary intake of polyvinyl chloride caused oxidative damage in *Dicentrarchus labrax* (European sea bass), leading to impaired immune activities (Espinosa et al., 2018). Barboza et al. (2018a) found that water with microplastics shows an increase in mercury levels in the liver and gills of *Dicentrarchus labrax*juveniles. Mercury and the microplastics, alone and in mixtures, caused oxidative stress in the liver and gills.

Zitouni et al. (2021) discovered the existence of various plastic additives in the tissues of *Dicentrarchus labrax*, such as plasticizers, fiber-reinforced plastics, curing agents, flame retardants, and heat stabilizers. The study confirmed the presence of 68 different additives, with the primary additive being an organic PVC stabilizer, followed by a DLC 785 lubricant with a diamond-like carbon coating. PEVA was found to be the most common tissue microplastic type, followed by PA, among the four polymer types identified by the researchers. In contrast, Alomar et al. (2017) observed no signs of oxidative damage in the *Mullus surmuletus’* liver after microplastic ingestion, indicating that the ingestion level was possibly too low to cause harm. However, they did detect a slight increase in GST activity, indicating that the low levels of microplastics may trigger the activation of the detoxification process in response to the low levels of microplastics (Table 1).

**TABLE 1:** Toxic effects of microplastics in fish.

Species	Microplastics				Organ affected	Toxic effect	References
	Type	Size	Colour	Concentration			
*Scophthalmus maximus*	Ethylene propylene	50–200 µm	Black and blue	-	Liver and gills	Gills contain more concentration of microplastics	Köktürk, et al. (2023)
						Oxidative damage mostly affected the liver and gills	
*Oreochromis mossambicus*	Polypropylene	-	-	100, 500, and 1,000 mg/kg	Liver	Fluctuations in homeostasis and increased ROS levels	Jeyavani et al. (2023)
						Higher apoptosis, DNA damage (genotoxicity), and histological changes	
*Danio rerio*	Propylene copolymer	-	-	0.1—1 mg/L	Brain, liver	Anxiety, ROS generation, mitochondrial dysfunction	Félix et al. (2023)
*Nothobranchius guentheri*	Polystyrene	5 μm and 15 μm	Blue	10 mg/L	Liver	Induced oxidative stress, reduced antioxidant and digestive enzymes, and hepatic dysfunction	Xia et al. (2020)
*Ctenopharyngodon idella*	Polystyrene	0.5 μm, 15 μm	-	100 μg/L, 500 μg/L	Liver	Induced oxidative stress and liver congestion	Hao et al. (2023)
						Altered gut microbiota and severe intestinal damage	
*Oryzias latipes*	Microfiber types microplastics	-	-	100 and 1,000 fibers/L	Liver	Increase in CAT, SOD, MDA and caspase-3	Kim et al. (2023)
						Induced apoptosis and DNA damage	
*Pseudobagrus fulvidraco*	Polyethylene	-	-	100, 200, 5,000 and 10,000 mg/L	Gut, gills and liver	Decreased RBC, Hb, haematocrit (Ht), calcium total protein and magnesium	Lee and Kim. (2023)
						Increased SOD, CAT and GST, decreased GSH	
*Sparus aurata*	Polystyrene	1–20 μm	-	25 and 250 mg/kg	Intestine	Increased ROS and MDA.	Del Piano et al. (2023)
						Altered SOD, CAT and GSH.	
						Upregulation of HSP70 and HSP90	
*Oreochromis niloticus*	Polyacrylamide	0.1–0.4 mm	-	0.018, 0.03 0.09 g/L	Gills, liver and intestine	Reduced CAT and GSH.	Raza et al. (2023)
						Increased MDA and lipid peroxidase levels	
*Danio rerio* and *Perca fluviatilis*	Polyethylene	10–45 μm and 106–125 μm	-	-	Liver and gills	Induced oxidative stress, DNA damage, lipid peroxidation and ubiquitination	Bobori et al. (2022a)
*Danio rerio* and *Perca fluviatilis*	Polypropylene	8–10 μm	-	1 mg/g and 10 mg/g	Liver and gills	Induced oxidative stress, DNA damage and apoptosis	Bobori et al. (2022b)
Xiphophorus helleri	Polystyrene	1 μm	-	-	Liver	Decreased antioxidant function, immunity, energy metabolism and growth performance	Zhang et al. (2022a)
						Weakened feed utilization	
Squalius squalus, *Blicca bjoerkna*, Capoeta umbla	47 microplastics	0–50, 50–100 µm	Black	-	Gastrointestinal tissues	Decreased SOD and CAT	Atamanalp et al. (2022)
*Capoeta trutta, Cyprinus carpio*						Increased ROS and MDA	
*Mugil cephalus*							
*Atherina mocho*							
*Sparus aurata*	LDPE	100 and 500 μm	-	-	Liver	Increased SOD, GRd, GST, MDA and caused oxidative damage	Capó et al. (2021)
*Gambusia affinis*	Polyethylene, polystyrene, polyvinylchloride, polyamide, and polycarbonate	-	-	-	Digestive tract and gills	Increased CAT, SOD and MDA	Buwano et al. (2022)
*Oreochromis niloticus*	Microplastics	>100 nm	White	1 mg/L, 10 mg/L and 100 mg/L	Liver	Decreased antioxidant capacity and increased ROS production	Hamed et al., 2020
Oryzias melastigma	Polystyrene	10 μm		2, 20, and 200 mg/L	Gill, intestine, liver, testis and ovaries	Increased CAT, GSH-PX and decreased GSH.	Wang et al. (2019)
						Changes in sex hormone levels	
*Cyprinus carpio*	PVC	-	White	45.55 μg/L, 91.1 μg/L, and 136.65 μg/L	Liver, intestine and gills	Induced oxidative stress	Xia et al. (2020)
						Decreased MDA and antioxidant activity	
*Carassius auratus*	Polyvinyl chloride	-	-	0.1 or 0.5 mg/L	Liver, intestine and gills	Increased GST, MDA, H_2_O_2_ activity and CYP1A expressions	Romano et al. (2020)
						Induced inflammation, hemorrhaging and necrosis	
*Oryzias javanicus*	Polystyrene	5 µm	White	100, 500 and 1,000 μg/L	Gut, liver, kidney and brain	Induced oxidative stress, lipid peroxidation neurotoxicity and inhibited AChE	Usman et al. (2021)

Fish is a valuable nutrient-rich food in the human diet. However, consuming microplastic-contaminated fish can be a potential health hazard, especially in areas where fish is a significant dietary staple or regions heavily polluted with these minute particles (Barboza et al., 2018b). In a recent study by Schwabl et al. (2019), the presence of microplastics was detected in human stool samples for the first time, suggesting that these particles are ingested and excreted by humans. The retention and elimination of microplastics within the body can be influenced by several factors, such as their shape, polymer type, size, surface charge, and chemistry, as well as any additional chemicals present in the microplastics ingested (Smith et al., 2018). Upon ingestion, the body can assimilate microplastics, and their cellular uptake can interact with proteins, phospholipids, and carbohydrates and cause adverse effects. Similar to nano-plastics, the absorption of microplastics is heavily dependent on their interactions with biological matter (Lehner et al., 2019). EFSA Panel on Contaminants in the Food Chain, 2016 has proposed that the human body can uptake only microplastics with a size lesser than 150 μm. Further investigation is needed to fully comprehend the mechanisms underlying microplastic absorption.

### 3.2 Pathways involved in microplastics-mediated oxidative damage

Microplastic exposure can trigger toxicity pathways, such as oxidative stress and inflammation. After ingestion, microplastics may affect a specific area or enter the blood and reach several organs and tissues. Moreover, microplastics bioaccumulate in the human body and exerts toxicological effects (Ferrante et al., 2022). Microplastics trigger ROS production through mitochondrial damage or by increasing the immune system responses while neutralizing harmful foreign particles (Yang et al., 2020). Microplastic contamination produces oxidative stress in proteins, lipids, and DNA by altering the antioxidant defense mechanisms (i.e., CAT, SOD, GST, GPx, and GSH) at the catalytic and transcriptional levels. In biological systems, microplastics deregulate the gene expression that controls oxidative stress. Thus, microplastics act as pro-oxidant stimuli, activating antioxidant gene expression through an Nrf2-dependent mechanism in marine vertebrates and invertebrates. These alterations are responsible for the induction of oxidative stress, neurotoxicity, immunological responses, endocrine system disruption, reproductive abnormities, genomic instability, embryotoxicity, and trans-generational toxicity (Alimba and Faggio, 2019).

Tang et al. (2018) observed that microplastic exposure in seabream heads altered the JNK (c-Jun N-terminal kinase) and ERK (extracellular signal-regulated kinase) signaling pathways which are involved in the detoxification process of fish. Polyvinylchloride and polyethylene exposure resulted in NRF2 upregulation, which led to oxidative stress in the renal primary leucocytes of seabream heads (Espinosa et al., 2018). Cocci et al. (2022) studied the polymers present in the gut of red mullet and the European hake as polyethylene and polypropylene. Microplastic abundance is positively correlated with antioxidant enzymes (catalase and superoxide dismutase) and cytokines (interleukin-1β, 10, and interferon) levels, causing ROS generation and immune cell infiltration in the gut. Their study found that microplastics affect fish gut through the induction of cytokine-dependent signaling pathways. Oxidative stress induced by microplastics mediates endoplasmic reticulum stress, leading to liver apoptosis. High concentration of polystyrene microplastic exposure in golden pompano (Trachinotus ovatus) caused oxidative damage and upregulation of genes (Grp78, Xbp-1, Eif-2α, and chop), resulting inendoplasmic reticulum stress (ERS). Severe oxidative stress raised the BAX/BCL-2 ratios and induced cell death (Yao et al., 2023).

Polystyrene microplastics affected the immune system of carp (*Cyprinus carpio*) and triggered the TLR2 signaling pathway. The study revealed that the mRNA and protein expressions of TLR2, COX2, TNF-α, tumor necrosis factor receptor-associated factor 6 (TRAF6), IL-1β, NF-κB, iNOS, and p65, were increased in hepatopancreas and hepatocytes of carp (Cui et al., 2023). Wang et al. (2022) found that extended exposure to polystyrene microplastics in loach juveniles (*Paramisgurnus dabryanus*) inhibited the expression of Keap1-Nrf2 signaling pathway genes in loach juveniles. Polystyrene microplastic exposure induced apoptosis by upregulating p53, gadd45ba, and caspase3b expressions. They also upregulated TNF-α and PTGS2A, which are important gene markers in the inflammatory mechanism in zebrafish (Umamaheshwari et al., 2021b). Qiang and Cheng. (2021) found that sub-chronic exposure to polystyrene in zebrafish resulted in enhanced ROS production in the ovary and testis, as well as lowered spermatocyte count. Additionally, male gonads showed an increase in apoptosis (BAX, p-53, caspase 7, 8, and 9) and a decrease in the thickness of the basement membrane. Long-term (35 days) exposure to polystyrene microplastics altered the metabolic pathways and gene regulatory patterns brought on by the production of ROS and oxidative stress in the same species (Umamaheshwarri et al., 2021a).

**FIGURE 4:**
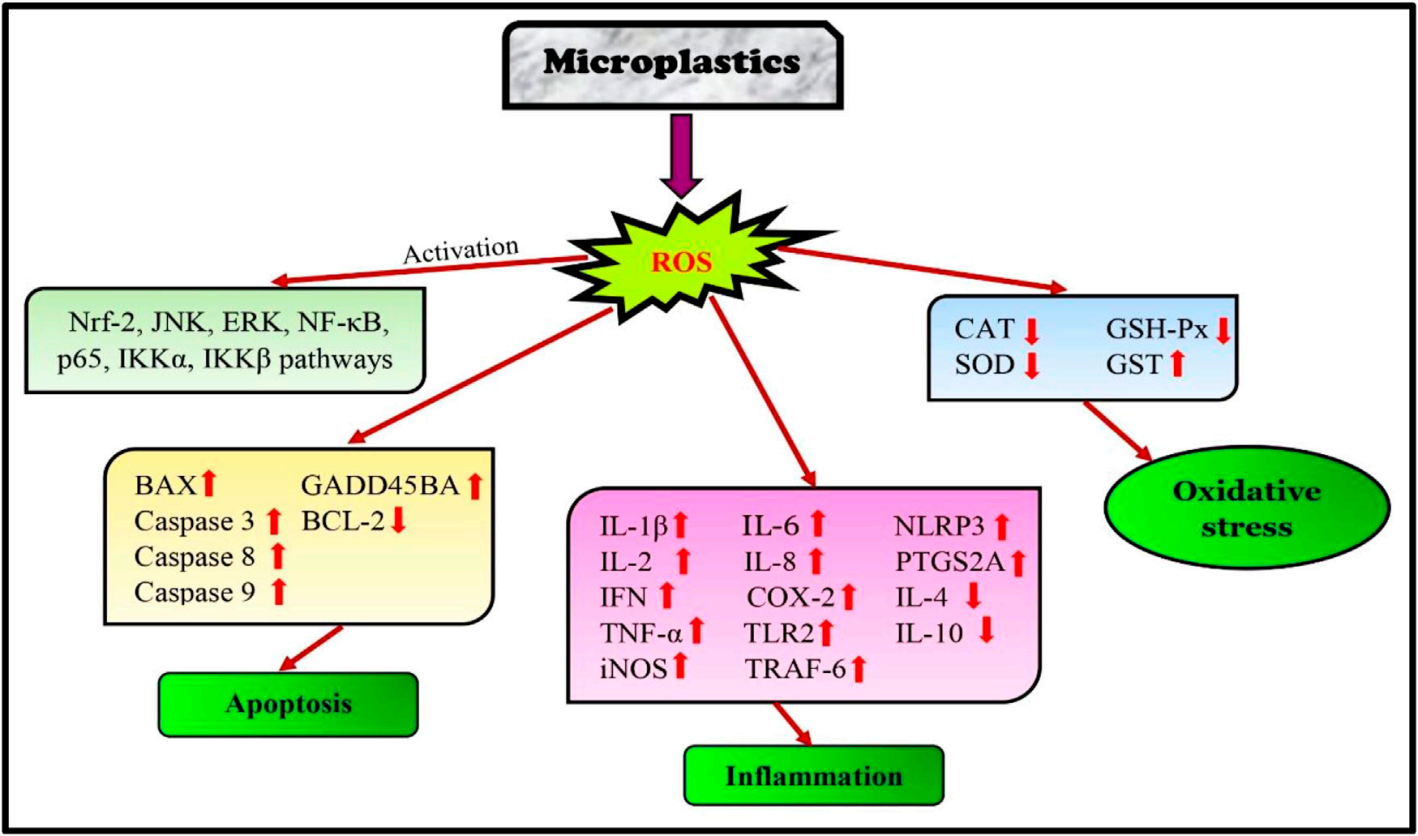
Pathways affected in fish due to microplastic exposure.

Polyethylene microplastics elevated the expression of BAX, p53, NF-κB, p65, IKKα, IKKβ, caspase-3, and 9 genes in the gills of carp. An important innate immunity element, NLRP3 (nucleotide-binding oligomerization domain-like receptor protein 3) inflammasomes was overactive. TNF-α, IFN-γ, IL-2, IL-6, IL-8, IL-1β, which cause immune disorders, increased, while IL-4 and IL-10, anti-inflammatory factors, decreased significantly. Thus, NF-κB pathway triggered oxidative stress, apoptosis, and NLRP3 inflammasome to promote inflammatory, immunological responses (Cao et al., 2023). Santos et al. (2022) studied the combined exposure of microplastics with copper (Cu) heavy metal in zebrafish (*Danio rerio*) gills. In the combination group (Cu25 + MPs), oxidative stress and lipid peroxidation were induced due to the inhibition of CAT and GPx activities in gills of zebrafish. Further, an upregulation of caspase-3 and tph1a (tryptophan hydroxylase 1a) genes and a downregulation of apoptosis and serotonin synthesis were also observed (Figure 4).

So, it can be summarized that the negative impacts of microplastics on fish growth and survival rates can have significant consequences for fish populations and the larger ecosystem. Reduced population sizes can affect aquatic ecosystems’ overall productivity and fish availability for human consumption.

## 4 Pesticides induced oxidative stress in fishes

The unregulated use of pesticides in aquatic environments and agriculture has led to their emergence as a significant threat to human health. Exposure to insecticides/pesticides, herbicides, and fungicides in marine and terrestrial ecosystems has resulted in various health issues among different animal species. These unintended consequences of pesticide use highlight the need for better regulation and control to minimize their adverse effects on the environment and living things. The use of pesticides in agriculture often leads to the dispersal of pollutants into surrounding soils, causing contamination that can seep into groundwater and eventually make its way into drinking water sources. Among aquatic creatures, fish are particularly vulnerable to the effects of these chemicals. Pesticide exposure can hinder their metabolism and even result in death, as has been shown in research by Huang et al. (2021).

Providing high-quality feed to animals is crucial for optimizing their performance and promoting their overall health and welfare (Walstra et al., 2005). Outcomes of good feeding results in the production of safe and quality of products of animal origin, mostly milk, meat and eggs. To meet the growing population and upliftment of the economy of the consumers, there is an increase in demand for animal proteins which leads to the intensification of livestock farming and use of commercial feeds (Foodmate, 2023). This increased the demand for selected grains (mostly maize), and different green as well as dry fodder (paddy straw and wheat straw), which indirectly increases the use of insecticides and pesticides to grow these crops as well as fodders in the limited available area. In developing countries such as India since its independence, the area under fodder production remains to be constant at the rate of 4% of cultivable land and other cereal crops growing are decreasing year after year, whereas milk production from that period has increased from 17.10 (MT) to 221. 00 (MT), egg production from 1832 million to 129,600 million, meat production from 1859.43 (‘000 tonnes, 1998–99) to 9,292.13 (‘000) tones in 2021–22 (Pib, 2023). It is believed that pesticide use has an important role in producing high amounts of feeds and fodders. Pesticides are classified into insecticides, herbicides, and fungicides, which cause various damages to fish population.

The global pesticides have been continually increased and estimated at 6 million tons of active ingredients annually. Worldwide herbicides, insecticides, and fungicides are the most commonly used pesticides. These residues should not pose health risks if they are below the exposure threshold known as maximum Residue Limits (MRL). For different products, different levels of tolerance have been accepted. For example, in milk, as many as 126 pesticides have been cleared by the registration committee for usage in some countries, including the Indian sub-continent. The source of contamination or main channels of pesticide flow to milk is feed, fodder, and contaminated utensils of milk collection. Fodders like paddy straw, wheat straw, maize, and beans are the major contributors to the residue in the milk. The tolerance values of commonly used pesticides in milk and milk products are presented in Table 2.

**TABLE 2:** Tolerance level of pesticide in milk.

Pesticide residue	Tolerance values (mg/kg)	
	Dairy products	Animal fat
DDT	1.0	1.0
Lindane	0.02	0.50
Trichlorphon	0.02	0.02
Dichlorphos	0.02	0.02
Butanol	0.02	0.5
Dimethoat	0.004	0.004
Carbaryl	0.02	0.02
Hexachlorbenzen	0.50	0.50

Similarly, meat and meat products also get contaminated with various pesticide residues, which affect the health of consumers. Mostly these pesticide residues are associated with feed production, parasitic treatments, processing, and packaging of meat and meat products. The pesticide residues in meat and its manufacturing readiness level are presented in Table 3.

**TABLE 3:** Tolerance level of pesticides in meat and their manufacturing readiness level.

Pesticide residue	Type of meat	MRL (mg/kg)
Chlorophyrifos	Cattle, Goat and Sheep	1.0
	Poultry	0.01
Carbaryl	Sheep, Goat and Cattle	0.05
	Poultry	0.05
Carbendazim	Cattle, Goat and sheep	0.05
	Poultry	0.05
Carbosulfan	Cattle, Goat and sheep	0.05
	Poultry	0.05
Carbofuran	Cattle, Goat and sheep	0.05
	Poultry	0.08

**FIGURE 5:**
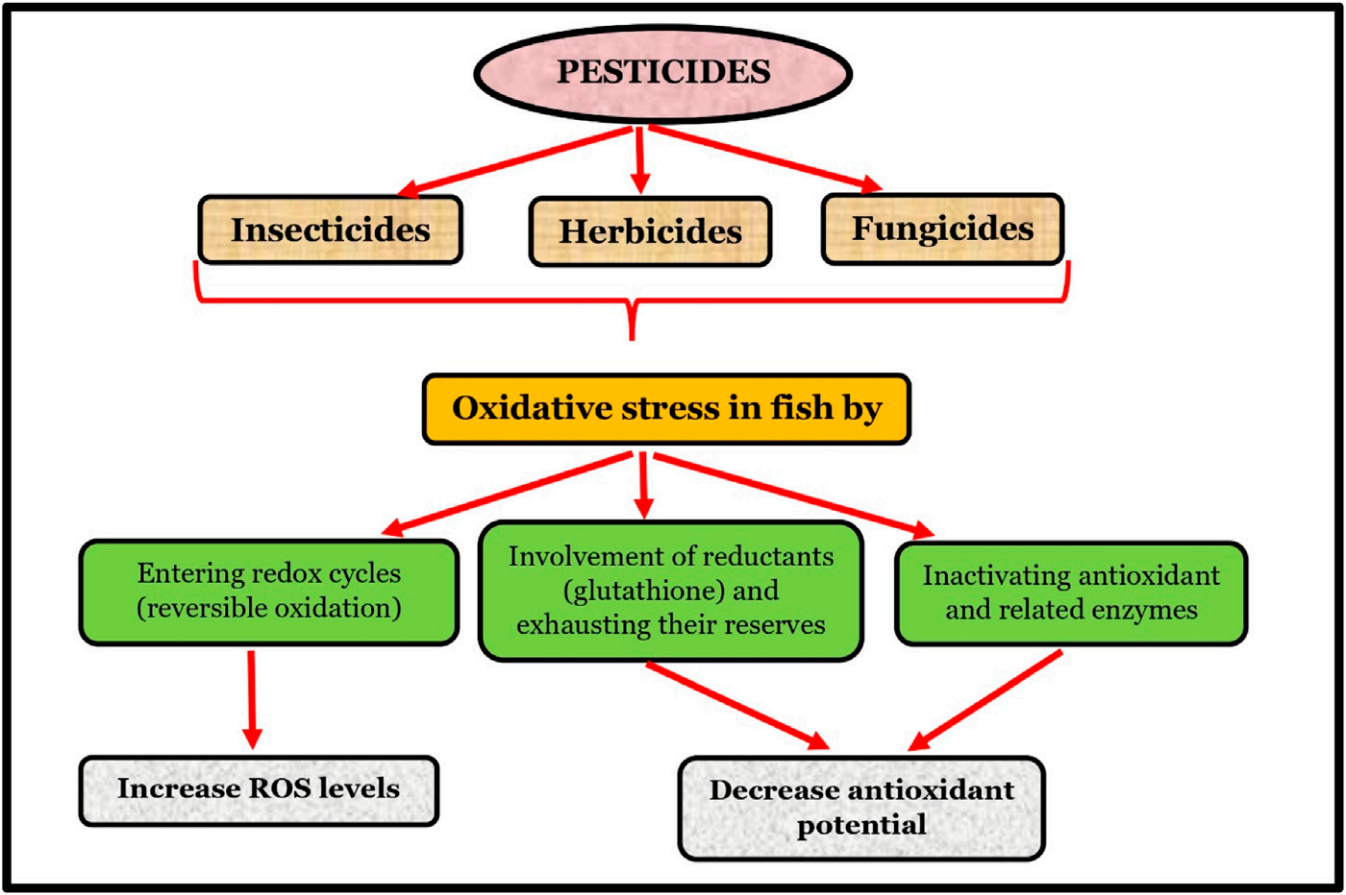
Pesticides in fish health.

Pesticides have the potential to produce ROS and induce oxidative damage in fish. A study by Shah and Marz. (2020) examined the histopathology and responses to oxidative stress in freshwater catfish and common carp found in the Ganga River in India, which is contaminated with pesticides. The study identified several abnormalities in the liver and gills obtained from the Rita & Carp from the Ganga River, contaminated with pesticides. The abnormalities included sloughing and disruption of the lamellar fusion, dilated vessels, lamellar epithelium, and enlargement of smooth muscle within the gill, as well as vacuolation in hepatocytes, necrosis, distorted arterial walls, and inflammation within the liver of both fish species. Mena et al. (2022) detected about 25 different pesticide residues, including fungicides, insecticides, and herbicides, in the fish *Astyanax aeneus*. Among pesticides, insecticide residues significantly influenced the fish, causing oxidative stress and neurotoxicity. Similarly, Ullah et al. (2018) identified malathion as a potent hepatotoxic presence of pesticides within the liver of Rohu fish (*Labeo rohita*). The liver was subjected to histopathological examination, which revealed a range of changes in the organ, including cellular swelling, hemorrhage, hepatic necrosis, vacuolation of glycogen and cells, fatty infiltration, and congestion (Figure 5).

### 4.1 Insecticides and fish health

Several studies have described that insecticide exposure in fish leads to oxidative stress. Imidacloprid and propoxur are insecticides that, in combination, induced oxidative damage and inhibited AChE activity in the neotropical fish, *Rhamdia quelen.* Increased catalase activity and altered antioxidant capacity against peroxides (ACAP) and thiobarbituric acid reactive substances (TBARS) confirmed the oxidative stress in the brain, gills, muscle and liver of the tested fish (Marins et al., 2021). Gonçalves et al. (2018) evaluated the oxidative stress biomarkers in the muscle, brain, gill and liver of *Astyanax jacuhiensis* in exposure to carbamate propoxur. There was an increase in the activity of GST in the liver and gills and protein carbonyl (PC) in the brain, whereas AChE activities were reduced in the brain and muscle. Similarly, combined exposure of lambda-cyhalothrin and imidacloprid in the gills and liver of streaked prochilod (*Prochilodus lineatus*) increased protein carbonylation and LPO level*.* Lambda-cyhalothrin was the most toxic insecticide, even at much lower concentrations than imidacloprid, causing DNA damage in the fish’s gills, liver, and brain (Alvim and dos Reis Martinez, 2019; Figure 5).

Pyriproxyfen (PPF) is a well-known synthetic insecticide used to control various insect species affecting crops in agricultural fields. Li et al., 2022a studied PPF exposure in *Labeo rohita* fish and found increased DNA damage in multiple tissues. The results showed increased oxidative stress indicators (ROS and TBARS) and antioxidant enzymes (GRd, SOD, peroxidase, and catalase) in several fish tissues. They recorded A decrease in the activity of different antioxidant enzymes in treated fish. Chlorpyrifos exposure in *Clarias gariepinus* elevated stress markers (glucose and cortisol), testosterone, luteinizing hormone, serum AST and ALT levels. CPF reduced total serum protein, lysozyme, follicle-stimulating hormone, AChE, albumin, estradiol hormone, and immunoglobulin activity levels while increasing hepatic DNA damage. CPF also induces oxidative stress in renal and hepatic tissues (Mansour et al., 2022).

Cypermethrin (CYP), a pyrethroid pesticide, increased the production of SOD, CAT, and malondialdehyde (MDA) in zebrafish embryos. In addition, changes in acetylcholine esterase, nitric oxide content, and Na^+^/K^+^-ATPase activity were observed. These changes disrupted cardiac development and ion regulation (Gupta et al., 2023). Acute deltamethrin exposure elevated malondialdehyde levels and lowered catalase enzyme. Initially, T-AOC and SOD levels were raised and then dropped. Being exposed to hypoxic conditions, deltamethrin exposure significantly reduced the survivability of crucian carp by lowering oxygen uptake, promoting lactate accumulation, and altering energy metabolism. Additionally, histological assays confirmed that deltamethrin caused apoptosis and gill damage under hypoxic conditions. Deltamethrin exposure induced oxidative and ER stress, which resulted in the impairment of hypoxic resistance of *Carassius auratus* (crucian carp) (Yuan et al., 2023). Phorate exposure to the kidney and liver of *Channa punctatus* caused an increase in the expression of apaf-1 (apoptotic peptidase activating factor-1), p53, and CAT*.* Thus, phorate-induced apoptosis, oxidative and DNA damage in *Channa punctatus* (Ratn et al., 2017).

### 4.2 Herbicides and fish health

Herbicides used in fields leach into nearby natural resources and bring about detrimental changes in aquatic life (Table 4). Acetochlor exposure in bighead carp increased DNA damage and resulted in oxidative damage. The cellular protein concentration was lower in gills, liver, brain, and kidneys of treated fish than in unexposed fish (Mahmood et al., 2022). Broadleaf weeds that compete with the nutrition uptake of agricultural crops are removed by continuous application of Pendimethalin, an herbicide. Pendimethalin causes toxic effects by disrupting the physiological and hematobiochemical reactions of fish. Exposure to pendimethalin in bighead carp (*Hypophthalmichthys nobilis*) will have a great impact on the physical and physiological activities of fish as reflected by abnormal behavior, loss of equilibrium, erratic swimming, air gulping, increased surface breathing, mucus secretion and fin tremors. The data showed that pendimethalin-exposed fish had increased serum LDH, ALT, and AST enzyme activity levels (Wang et al., 2022).

**TABLE 4:** Toxic effects of pesticides in fish.

Species	Pesticides			Organ affected	Toxic effect	References
	Type	Name	Concentration			
*Cyprinus carpio*	Insecticide	Abamectin	3.005 and 12.02 μg/L	Gills	Induced oxidative stress, respiratory toxicity, inflammation and apoptosis	Feng et al. (2023)
					Inhibited autophagy by activating PI3K/AKT/mTOR pathway	
*Clarias gariepinus*	Herbicide	Glyphosphate based herbicide	0.02–1 mg/L	Gills, liver and brain	Reduced GSH and SOD.	Kale et al. (2023)
					Increased MDA.	
*Clarias gariepinus*	Herbicide	Fluazifop-p-butyl**(**FPB)	1.80, 3.50, and 7.10 mg/L	Blood, liver and gills	Decline in SOD, CAT and GPx	Anih et al. (2023)
					Increase in MDA, GSH, GRd	
*Clarias batrachus*	Herbicide	Pendimethalin	3.55 mg/L	Liver	Increased SOD and CAT.	Gupta and Verma. (2022)
					Altered lipid peroxidation and anti-oxidant enzyme activity	
*Oreochromis niloticus*	Herbicide	Glyphosphate	0.6 mg/L	Liver	Increased ALT, AST, cortisol and MDA levels	Abdelmagid et al. (2022)
*Danio rerio*	Herbicide	Oxyflourpen	0.4, 0.8, 1.2 mg/L	Kidney	Induced ROS generation, apoptosis and nephrotoxicity	Huang et al. (2022)
*Jenynsia multidentata*	Insecticide	Cypermethrin, Chlorpyrifos	0.4 μg/L	Intestine, liver, gills and muscle	Increased GST	Bonansea et al., 2017
*Rhamdia quelen*	Insecticide	Imidacloprid	0.11 μg/L	Gills, liver, brain and muscle	Inhibited AChE, induced oxidative stress	Marins et al. (2021)
		Propoxur	0.039 μg/L			
		Mix	0.11 μg/L + 0.039 μg/L			
*Oreochromis niloticus*	Insecticide	Chlorpyrifos	1–150 μg/L	Liver, spleen and heart	Increased GST, SOD and CAT	Farhan et al. (2021)
*Astyanax altiparanae*	Insecticide	Imidacloprid	0.07, 0.63, 5.94 and 53.95 μg/L	Muscle, gills	Increased LPO and decreased AChE	Almeida et al. (2021)
					Induced genotoxicity	
*Cyprinus carpio*	Fungicide	Difenoconazole	-	Heart	Increased MDA, CAT, SOD and GSH-Px	Wang et al. (2022c)
					Induced inflammation, apoptosis cardiotoxicity and autophagy	
*Danio rerio*	Fungicide	Tebuconazole	-	Liver	Induced ROS generation	Li et al. (2020a)
*Oreochromis niloticus*	Insecticide	Fenitrothion	0.20 mg/L	Gills, kidney and liver	Increased GST and SOD, inhibited AChE	Fouad et al. (2022)
		Thiobencarb	0.32 mg/L			
*Cyprinus carpio*	Insecticide	Cypermethrin	0.0002, 0.0003 & 0.0006 μg/L	Gills and liver	Altered behavioural patterns	Yancheva et al. (2022)
		Chlorpyrifos	0.03, 0.05, & 0.10 μg/L			
*Danio rerio*	Insecticide	Imidacloprid	0.15, 15, and 45 μg/L	Brain	Increased GST and reduced AChE and protein carbonyl	Guerra et al. (2021)
*Oreochromis niloticus*	Insecticides	Chlorpyrifos	480 g/L	Liver	Altered CAT levels, decreased SOD	Firat and Tutus. (2020)
		Avermectin/Abamectin	18 g/L		Increased GSH and MDA	
		Emamectin benzoate	50 g/L			
*Australoheros facetus*	Insecticide	Imidacloprid	100 and 1,000 μg L	Brain, gills and liver	Induced oxidative stress	Iturburu et al. (2018)
*Cyprinus carpio* and *Danio rerio*	Herbicide	Glyphosphate	0.05–50 mg/L	Embryo	Induced acute toxicity	Fiorino et al. (2018)

Glyphosate-induced neurotoxicity and increase in anxiety in adult zebrafish. There was an increase in dopamine and serotonin levels. Downregulation of gene expression in the dopaminergic system, namely th1, th2, comtb, and scl6a3, was observed along with the elevated levels of enzymic antioxidants, CAT and SOD, which led to a decline in the glutathione reserves. All these responses evoked in the antioxidant defense mechanisms, and these results were also due to oxidative stress and lipid peroxidation observed in the fish brain (Faria et al., 2021). Dicamba (DIC) and 2,4-dichlorophenoxyacetic acid (2,4-D) are auxinic herbicides used to control weeds in agriculture. DIC and 2,4-D exposure induced oxidative damage at purine levels in *Cnesterodon decemmaculatus*. Herbicide-exposed fish revealed increased CAT and GST activities and decreased GSH content. Additionally, diminished AChE activity and total protein content were observed (Es Ruiz de Arcuate et al., 2019)*.*

### 4.3 Fungicides and fish health

Fungicides are most widely and extensively preferred among the various agricultural pesticides. Amidst the diversified range of fungicides available, difenoconazole is generally used triazole fungicide in the production of agricultural crops. In carp, difenoconazole exposure might cause ROS production resulting in oxidative stress with increased MDA levels and subsequent decline in the activity level of enzymatic antioxidants such as CAT, SOD, and GPx (Wu et al., 2023). Nataraj et al. (2023) studied the effects of difenoconazole exposure in *Labeo rohita.* Among the various organs of *Labeo rohita,* such as gill, liver and kidneys examined for their antioxidant efficiencies, a significant reduction in the activities of SOD and CAT was observed in comparison with the control fish. In addition, GST activity and LPO level were found to be more in the vital tissues of the carp. A drastic change in the gill histology of the carp due to difenoconazole exposure caused hypertrophy, epithelial lifting, epithelial necrosis, and lamellar fusion. Furthermore, the presence of pyknotic nuclei was a significant and noticeable change in both the liver and kidney. Further, dysfunctions like cellular edema, shrinkage of glomeruli, vacuolation, and tubular necrosis occurred in the liver of *Labeo rohita*. Obvious DNA damage with prominent tail formation occurred in a time and concentration-dependent manner. Crupkin et al. (2021) reported that azoxystrobin fungicide caused genotoxicity and oxidative stress in *Australoheros facetus.* In conjunction with this, inhibition of SOD occurred in the liver and gills of juvenile fish.

The global use of folpet as a fungicide has been widespread for the past 50 years. Folpet exposure in common carp (*Cyprinus carpio*) for 14 days showed a significant reduction in RBC, hemoglobin levels, hematocrit values, and increased activity of antioxidant enzymes (CAT, SOD, GPx) along with HSP70 genes. Besides its impact on the blood cells, folpet also causes DNA damage and oxidative stress (Acar et al., 2023). Pyraclostrobin (PYR), a strobilurin fungicide, poses a potential risk to aquatic organisms as it promotes ROS and MDA content in hepatopancreas of organisms. PYR-exposed aquatic lives showed enhanced expressions of the genes *p38, JNK MAPK, HSP70*, and *HSP90*. Remarkable reduction in the antioxidant enzymes and biomarkers levels, upregulation of BAX, APAF-1, Caspase-3, and 9, and downregulation of BCL-2 in carp hepatopancreas upon exposure to pyraclostrobin. Inflammatory response of the exposed fish showed changes in the IL-1β, IL-6, TNF-α, TGF-β, NF-κB, and p65 and immune parameters (IgM, LYZ, ACP, C3, and AKP), indicating that PYR exposure caused an induction in apoptosis due to oxidative stress and also evoked inflammation and immunotoxicity in common carp (Zhao et al., 2022).

The above-cited studies reveal that pesticides entering aquatic environments affect fish health by inducing oxidative stress, immunotoxicity, and inflammatory responses. Pesticides also cause behavioral changes and organ damage in fish. This affects fish’s growth, reproduction, and nutritional quality, making it a non-profitable and non-consumable product. All these changes depend upon the pesticides’ exposure duration, concentration, and chemical structure. Aquatic systems may contain pesticides as a result of anthropogenic or natural causes. Moreover, consuming pesticide-contaminated fish has a direct impact on human health. Fish containing pesticide residues has the potential to cause health problems in humans, such as epilepsy, somatic growth dysfunction, cancer, genetic damage, liver and kidney dysfunction, leukemia, decreased fertility, teratogenesis, and suppression of the immune system (Hoque et al., 2022). Some studies reported that the consumption pesticide contaminated fish does not affect human health much. However, potential long-term exposure can result in causing various ailments in humans (Chang et al., 2020; Tyohemba et al., 2021).

### 4.4 Pathways involved in pesticide-mediated oxidative damage

Pesticides can cause oxidative stress and carcinogenesis in fish. Long-time exposure to malathion pesticides in fish increased the expression of tp53 and its related genes, which in turn activated theatm/atr gene expression. The *hif-1* gene showed an increase in expression, but the Ras (a proto-oncogene) was unaffected. The continuation of this condition downregulated BCL2 levels, and the BCL2/bax ratio resulted in an apoptotic response (de Souza et al., 2023). In grass carp (*Ctenopharyngodon idellus*), the neonicotinoid chemical imidacloprid (IMI), which is commonly used in agricultural production, induced mitochondrial dysfunction, which led to inflammation and an increase in NF-kB, TNF, IL-1, and IL-6 (pro-inflammatory genes) expression. IMI exposure triggered apoptosis by encouraging the release of Cyt-C (cytochrome-C), BCL-2 downregulation, and BAX, Caspase 3 and 9 upregulation. IMI exposure also caused the expression of BNIP3, LC3B, and P62 (mitophagy-related genes). The study results reveal IMI-induced inflammation, mitophagy, oxidative damage, and mitochondrial apoptosis in the hepatocytes of grass carp through the NF-kB/JNK pathway (Miao et al., 2022).

In carp, avermectin disrupted the blood-brain barrier and promoted apoptosis, inflammation, neurotoxicity, and oxidative damage involving the NF-κB and PI3K/Akt signaling pathways. Avermectin also increased IL-1β, IL-6, TNF-α, and iNOS, resulting in brain inflammation (Zhang et al., 2022). Difenoconazole (DFZ) is a long-lasting fungicide in the marine environment. DFZ altered the expressions of p53, FAS, BCL-2, BAX, Caspase3, 8 and 9. SOD, CAT, and GPx were down-regulated, and the NF-κB signaling pathway was activated in the spleen tissue of *Cyprinus carpio*. An increase in Il-6, Il-1β, and TNF-α (pro-inflammatory cytokines) and a subsequent decrease in Il-10 and TGF-β1 (anti-inflammatory cytokines) were observed. DFZ exposure damaged the carp’s spleen tissue by inducing oxidative stress, apoptosis, inflammatory responses, and immunosuppression (Liu et al., 2022). Enrofloxacin is a type of fluoroquinolone that has high risks to aquatic organisms. The effects of ENR exposure in *Pelteobagrrus fulvidraco’s* (yellow catfish) gills*,* reduced Na^+^/K^+^-ATPase activity and impaired immune system. Meanwhile, ENR induced oxidative stress, apoptosis, and MAPK signaling at higher concentrations (Xu et al., 2022).

**FIGURE 6:**
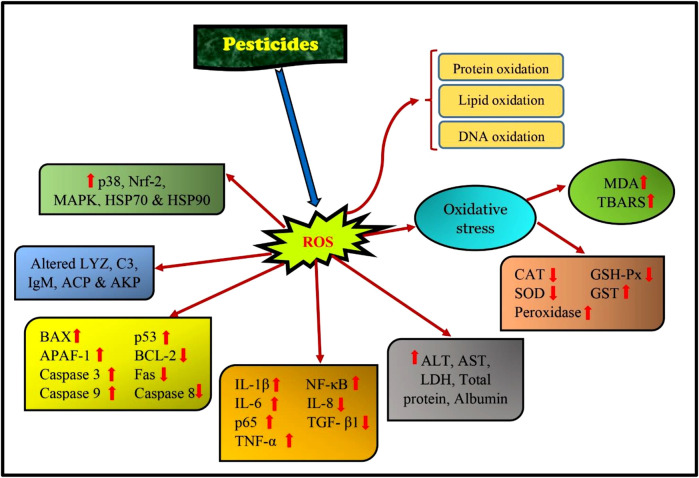
Pathways affected in fish due to pesticide exposure.

**FIGURE 7:**
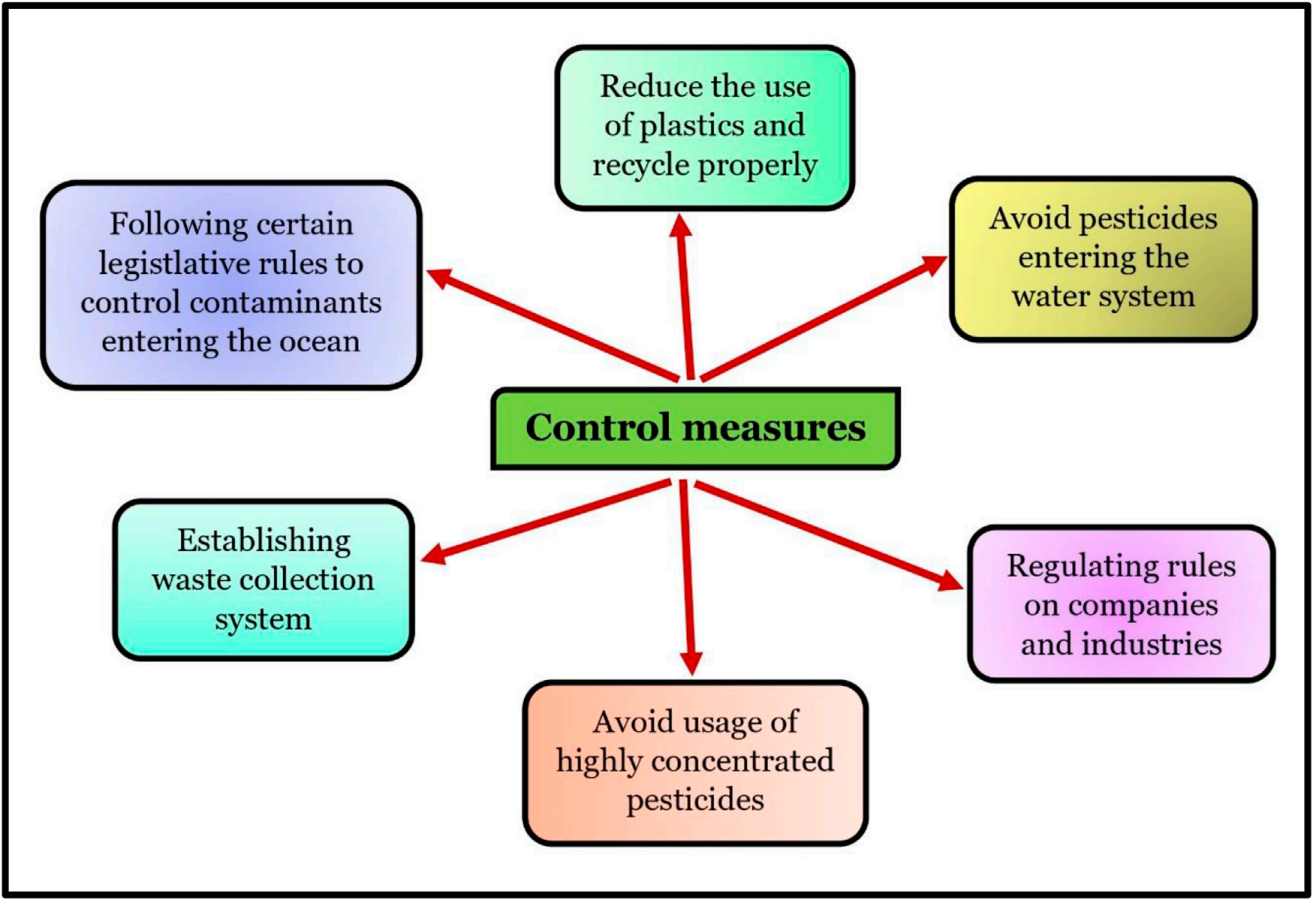
Control measures for contaminants entering the ocean.

In common carp kidneys, chlorpyrifos inhibited the miR-19a, which enhanced AMPK (AMP-activated protein kinase). Chlorpyrifos exposure enhanced the expression of apoptosis and autophagy-related genes such as TSC complex subunit 2 (TSC2), BAX, light chain 3 (LC3), p53, Dynein, Caspase-3, and 9. Exposure to chlorpyrifos reduced the expressions of Rheb (Ras homolog mTORC1 binding), BCL-2, and mTOR (mechanistic Target of Rapamycin). Thus, CPF promoted autophagy, oxidative damage, and apoptosis in the experimental fishes more than the control fish group (Zhang et al., 2019). Zhao et al. (2021) found that CPF exposure in largemouth bass (*Micropterus salmoides*) caused an increase in IL-8, TNF-α, and IL-15 and a decrease in TGF-β1 and IL-10. CPF exposure also promoted overexpression of Caspase-3, 8, 9, and BAX which resulted in apoptosis. According to Li et al. (2022c), using acetochlor herbicide on grass carp caused an increase in the levels of BAX, Beclin1, LC3B, P62, and Caspase 3 expression while reducing the expression of BCL2. Additionally, the study revealed a rise in ROS levels and a decrease in PPAR/RXR pathway expressions following acetochlor treatment. As a result, acetochlor exposure led to a cascade of apoptosis via the BCL2/BAX/Casp3 pathway and Beclin1-dependent autophagy, which was triggered by ROS-mediated PPAR/RXR inhibition. TNF-α, Il-6, Il-1β, and iNOS were increased with the use of difenoconazole fungicide, while the transcription level of TGF-β1 and Il-10 was inhibited. According to Wu et al. (2023), using the product led to an increase in BAX, p53, Caspase-3, and 9. Meanwhile, the BCL-2, Capsase-8, and FAS were inhibited. Furthermore, there was a noticeable increase in LC3II protein expression levels and the atg5 (autophagy-related gene), while the transcript levels of p62 were found to be decreased. This suggests that the use of the product stimulated autophagy (Figures 6, 7).

Li et al. (2021) studied how both cypermethrin (CMN) and sulfamethoxazole (SMZ) in combination affected grass carp. They revealed that the first 30 genes controlled by CMN and SMZ were involved in pathways that were closely related. Moreover, the combined exposure group experienced greater imbalances in oxidative stress index than the single exposure group. In addition, activation of the NF-κB signaling resulted in an immuno-inflammatory response in the combined exposure. Feng et al. (2023) reported exposure to abamectin pesticide-induced respiratory system toxicity in carp by activating the PI3K/AKT/mTOR pathway and inhibiting autophagy, resulting in oxidative stress, inflammation, and apoptosis; Yuan et al. (2023) found that high concentrations of deltamethrin insecticide led to the downregulation of Nrf2 signaling and its related genes (CAT, SOD, and GPX1) and the upregulation of KEAP1 gene. In addition, deltamethrin resulted in the activation of the IRE1 and PERK-ATF4-CHOP signaling pathways, leading to endoplasmic reticulum stress in crucian carp. According to Li et al. (2019), treatment with GBH (glyphosate-based herbicide increased the levels of HSP70, HSP90, and HSC70 in various organs of fish, suggesting a possible link between HSP induction and GBH toxicity. Further investigation showed that GBH notably increased IL-1β, IL-6, IL-8, IFN-γ, and TNF-α expression and altered the IL-10 and TGF-β levels. GBH exposure also inhibited T-AOC activity but increased the MDA level. These results suggest that GBH induces oxidative stress and immuno-toxicity in common carp (Figure 6). So, in general, several pesticides have been found to have adverse effects on the fish population and prevalent oxidative stress in them seems to be common under pesticide exposure.

## 5 Combined effects of microplastics and pesticides on fish health

The combined effects of microplastics and pesticides on fish health have yet to be explored much due to their complicated interactions (Brandts et al., 2018; Burgos-Aceveset al., 2021). Recently, Hannachi et al. (2021) and Karbalaei et al. (2021) investigated the effects of polystyrene microplastics in combination with chlorpyrifos insecticide in *Onchorhynchus mykiss* (rainbow trout). The results reveal that polystyrene and cholpyrifos together lowered the nutritional value, increased toxicity, and caused necrosis and inflammation in the fish. Li et al. (2022b) studied the impacts of combined polystyrene and difenoconazole that increased the oxidative stress in the liver of zebrafish. The presence of microplastics has alleviated the toxic effect of difenoconazole, which resulted in altered gene expressions. Polystyrene microplastics, along with imidacloprid pesticide altered the glycolipid metabolism and caused oxidative stress and inflammatory response in zebrafish liver (Luo et al., 2021). From the above-reported studies, it can be confirmed that microplastics, in combination with pesticides, have a definite impact on fish health by causing oxidative stress and affecting their nutritional values (Li et al., 2018). However, to gain more knowledge in this area of research, the mechanism behind the combined effect of microplastics and pesticides must be explored in the future (Garrido et al., 2020; Frank et al., 2022).

Concurrently, it is imperative to administer effective management strategies to mitigate oxidative stress and disease susceptibility in fish to optimize their utilization. Many conventional techniques and medicines are used to tackle several diseases and stress in fish population. However, the use of modern technology always seems to have better output than conventional technologies. As a matter of fact, more than contemporary technology of adapting the conventional stress and disease management in fish is required. So, the focus must be given to modern technologies such as nano-technology for fish stress and disease management.

## 6 Nano-forms of chemicals for fish health and disease management

It was found that the nano-formulations of various chemicals induced oxidative stress in fish. For example, ZnO nanoparticles are found to increase the toxic load in the liver of teleost fish via mitochondrial-dependent pathways. More particularly, the above nanoparticle induces lipid accumulation, low lipolysis and activates mitophagy (Chen et al., 2022). Copper ferrite nanoparticles increase LPO, GST, and GPx and alleviate CAT and GSH level, and are finally considered cytotoxic in the ovaries of catfish (Srikanth and Nutalapati, 2022). Maghemite (gamma-Fe_2_O_3_) nanoparticles at 40 and 60 ppm hampers developments and induces oxidative stress in Zebrafish Embryos/Larvae (Thirumurthi et al., 2022). Another metallic nanoparticle, namely magnetic nanoparticles, is noted to be neurotoxic in nature and induces oxidative stress by inhibiting the activity of SOD, CAT, GPx, and GSH values in the brain of rainbow trout (Ucar et al., 2022). Polystyrene nanoforms are not only responsible for generating oxidative stress by stimulating LPO generation but also induce cardiotoxicity in carps (Wu et al., 2022). Nano-forms of various plastics damage the reproductive cells and increase oxidative stress in Zebrafish, as documented by Sarasamma et al. (2020). Similarly, some other non-metal nanoparticles such as graphene oxide in zebrafish (Chen et al., 2016), in *Anabas testudineus* (Paital et al., 2019), fullerene in freshwater fish *Carassius auratus* (Zhu et al., 2008), pyrethroid nano-forms in carp, tilapia, and trout (Yang et al., 2020) are proved to induce oxidative stress, primarily by deregulating the antioxidant enzymes.

Since fish is regarded as an important source of protein, essential fatty acids, vitamins, and minerals such as selenium and iodine that are usually absent in meat or other crops, stress and disease management are very important. As with most aquatic organisms, cultured fish species around the world experience a variety of infectious and non-infectious diseases due to contaminated water from industrial and agricultural sources and intensive aquaculture. Additionally, because fish in captivity have a reduced immune system, stress on them increases the spread of diseases like bacteria, fungi, and viruses. Fish infections have so received much attention in recent years. Due to illnesses brought on by numerous infections, the aquaculture sector experiences significant losses each year. Therefore, it is crucial to effectively detect and manage diseases in order to maximize productivity and guarantee the high quality of the finished product (Ninawe et al., 2017).

### 6.1 Role of nanoparticles in disease diagnosis

The potential uses of nanotechnology in the aquaculture and fisheries sectors are numerous. Typically, a nanoparticle (NP) is defined as a structure with a size between 0.1 and 100 nm (1/1,000,000 mm). Nanoparticles have attracted much attention in diagnosing various fish diseases due to their sensitiveness and preciseness in diagnosing various bacterial, viral and fungal diseases (Munawar et al., 2021). Various kinds of nanoparticles, like ZnO, TiO_2_, Cu-based nanoparticles, silver nanoparticles, etc, are used in the diagnosis of fish diseases. Ravikumar et al. (2012) reported that nanoparticles of CeO_2_ could be used for control of several fish diseases associated with *Vibrio parahaemolyticus*, *Serratia spp*., *Aeromonas hydrophila, Vibrio harveyi, Bacillus subtilis,* and *Serratia sp*. Saad et al. (2022) investigated the effects of biological selenium nanoparticles on bacterial load and heavy metal accumulation in Nile tilapia fish and reported that those nanoparticles could able to reduce the load of *Aeromonas hydrophila* by 28%–45% and cadmium and mercury levels by 50%–87% and 57%–73%, respectively in fish organs. Chen et al. (2016) reported that nano-biosensors can detect fish contaminants and very low concentrations of pathogens such as bacteria, viruses, and parasites. They may be based on various nanomaterials, such as carbon nanotubes.

### 6.2 Role of nanoparticles in water treatment

In addition to this, the microorganisms and heavy metals present in the watercourse retards the growth rate of fishes leading to a great economic loss in the fishery sector (Shah and Praveen, 2020). Nanotechnology has been used very effectively to remove toxins from water. Poor performance and harm to the organisms result from water quality loss (Boyd & Tucker, 2012). Globally, the use of nanotechnology in water treatment is on the rise, with a wide range of potential customer-specific uses. The capacity to include diverse qualities in multifunctional materials is one of the most beneficial features of these systems, including nano-adsorbents, nanometals, nanomembranes, and nano-photocatalysts, among others. For instance, nanomaterials can simultaneously remove particles and pollutants and increase process efficiency (Gehrke et al., 2015). Water treatment is one of the most crucial foundational elements needed for sustained aquaculture. The excessive use of antibiotics and other synthetic chemical substances in fisheries and the discharge of waste materials from cities, businesses, and agriculture, have made water poisoning leading to an indirect impact on aquatic individuals as well as causing foodborne illnesses when consumed by human beings. These waters contain heavy metals that cause fish to grow more slowly and eventually die. Aquaculture uses nanotechnology mostly for treating water to provide a suitable environment for fish growth and reproduction. In this light, the scientific community supports adsorption and photocatalysis as the most effective and reasonably priced methods of water purification. Activated forms of carbon or alumina, along with either zeolite or iron-containing materials can be used effectively to hold both aerobic and anaerobic biofilm to remove various pollutants like nitrates, nitrites and ammonia.

When silver nanoparticles are used to cure fungi in water directly, young trout have been found to be affected, although rainbow trout fish kept in fish farms may not become infected by fungi if their water is filtered through a device coated with silver nanoparticles. The ultrafine form of iron particles can be used as a cleaning agent for less harmful compounds present in water courses like dioxins, carbon tetrachloride, trichloroethane, or polychlorinated biphenyls (Rather et al., 2011). TiO2 has been used effectively to treat wastewater due to its non-toxicity property, biological and chemical stability and photocatalytic activities. Many previous studies have documented the photocatalytic activities of TiO2 against various disease-causing bacterial, algal, and viral agents (Ouyang et al., 2016). Kuang et al. (2017) reported that graphene oxide and graphene nanosheets can be used as efficient water source cleaning agent. The graphene oxide -TiO2 combination has been used for removing various heavy metal ions and organic dyes from polluted water (Atchudan et al., 2017). Also, silver nanoparticles are gaining much more importance in cleaning the water source that is used for fish rearing due to their advanced cleaning system (Munawar et al., 2021).

### 6.3 Effects of nanoparticles on fish health and growth

Unquestionably, nutraceuticals are recognized to be highly effective in enhancing fish development and immune characteristics. However, the costs associated with their incorporation are higher than the minimum requirements. Many studies supported the use of nanotechnology in fisheries for the efficient delivery of nutraceuticals and dietary supplements. By making nutrients more soluble and protecting those from the harsh gut environment, these systems primarily aim to increase the bioavailability, bio-accessibility, and, subsequently, effectiveness of the nutrients. Previous studies also reported a faster growth rate and higher final body weight in *Carassius auratus gibelio* young carp when fed with diet supplemented with iron and selenium nanoparticles (ETC, 2003; Frederick et al., 2010). Iron nanoparticles have been proven to have a growth-promoting effect on young carp and sturgeon. Additionally, it was discovered that adding nano-selenium to a fish’s diet could increase its weight, relative growth performance, antioxidant activity, glutathione peroxidase activity, and concentrations of selenium in muscle of crucian carp (*C. auratus gibelio*) (Bhupinder, 2014). Khosravi-Katuli et al. (2017) reported that using nanoparticles in the fisheries sector enhanced immunomodulation, digestion and reproduction compared to other forms of Se. Wang et al. (2013) also reported that nano supplementation could help in increasing fish and animal productivity by reducing oxidative stress. Previous studies reported that diets supplemented with 0.2 mg/kg selenium nanoparticles had rapid weight gain rate and a reduced feed coefficient than feed with 0, 0.1, 0.4, 0.8, and 1.6 mg/kg selenium nanoparticles (Qin et al., 2016). Feed supplemented with selenium nanoparticles increases immunity and disease resistance while providing resistance to the effects of hypoxic stress (Kumar et al., 2017).

When delivering biological materials, nanoparticles are less expensive than other materials. Additionally, nanoparticles possess adjuvant qualities that can raise antigen effectiveness. The cellular uptake of nanoparticles is facilitated by their diminutive size, as they are only capable of entering living cells via cellular endocytosis (Zhao et al., 2014). Adjuvants lessen administration frequency while enhancing immune response (Evensen, 2009). Luis et al. (2019) reported that the stability, solubility, targeting, biocompatibility, and permeability of vaccines can be enhanced by the application of nanotechnology.

In conclusion, it can be asserted that the implementation of nanotechnology in aquaculture systems, the administration of pharmaceuticals through porous nanostructures in fish feed, and the utilization of nano sensors for pathogen detection in aquaculture systems hold promising prospects for impacting fish health.

## 7 Conclusion

The health and nutritional quality of various marine organisms, especially fish, have been compromised due to contaminants like microplastics and pesticides. Fish provide many benefits to humans, including food and economic benefits. Microplastics and pesticides alone or in combination with other environmental contaminants affect fish health in various ways. Toxic nano-materials were also found to impart (oxidative) stress in fish. Exposure to these contaminants in fish causes oxidative stress, immunotoxicity, neurotoxicity, inflammation, organ damage, physical injury, reduced growth, and behavioral alterations. Moreover, microplastic and pesticide residues in the aquatic organism may transfer through food webs and may cause harmful effects on human health. This review provides a clear insight into microplastics and pesticides causing oxidative stress and affecting the signaling pathways in fish. While research in this field has increased in the last few years, much is still not fully understood about the prolonged effects of microplastic exposure on aquatic organisms and human health. Studies have suggested that microplastics may accumulate in human tissues and organs, potentially leading to health problems such as oxidative stress, inflammation, and damage to the organs such as the liver, kidneys, brain, and gut. However, further investigation is necessary to attain a comprehensive understanding of stated hazards and develop strategies to mitigate potential exposure. The application of nano-technology in the fisheries industry has been proposed as a potential solution to address various challenges, particularly those associated with fish diseases. Future research should include comparative and extended monitoring studies to evaluate the presence of microplastics and pesticides in fish that enter food webs, as well as their potential negative impacts on individual health and overall quality of life throughout their lifespan.
